# Use of Generative Artificial Intelligence in the Management of Low Back Pain: a Scoping Review

**DOI:** 10.1007/s10916-026-02406-0

**Published:** 2026-05-09

**Authors:** Renjie Tu, Simon French, Isaac Searant, Jae Woo Chung, Mark Hancock, Farah Magrabi, Aron Downie

**Affiliations:** 1 Department of Chiropractic and Spinal Pain Research Centre, Macquarie University, Sydney, NSW Australia; 2 Department of Health Sciences and Spinal Pain Research Centre, Macquarie University, Sydney, NSW Australia; 3 Centre for Health Informatics, Australian Institute of Health Innovation, Macquarie University, Macquarie Park, NSW Australia

**Keywords:** Generative artificial intelligence, Low back pain, Scoping review, ChatGPT, Decision support systems, Patient education

## Abstract

Background Context. Low back pain (LBP) is common and a major cause of disability globally. Generative artificial intelligence (GenAI) such as ChatGPT may have the potential to improve LBP, but it is unknown how GenAI is being used in clinical care settings. Purpose. This scoping review aimed to map and synthesise the existing literature on the use of GenAI in the management of LBP across all settings. Study Design. This review followed the scoping review methodology of the Joanna Briggs Institute and Preferred Reporting Items for Systematic Reviews and Meta-Analyses extension for Scoping Reviews (PRISMA-ScR) guidelines. Methods. We searched the Cochrane Library, Medline, Scopus, Embase, Web of Science, CINAHL, and preprint platforms for studies published between January 2012 and July 2025, on the use of GenAI in the management of LBP across all healthcare settings. The included studies were categorised according to the primary purpose of GenAI implementation. Results. A total of 31 studies were included. Seventeen studies evaluated GenAI as a potential support tool for diagnosis and treatment, including answering specific questions about LBP (n = 8), generating advice from clinical cases (n = 4), and generating advice from diagnostic imaging (n = 5). Eight studies examined the use of GenAI to provide educational advice for patients. Other uses included research support (n = 5) and clinical documentation support (n = 1). GenAI advice for the management of LBP demonstrated higher agreement when using simpler tasks, more detailed prompts, or newer generation models. Reported errors in GenAI advice included inconsistency with guidelines or clinicians’ advice, hallucination, low accuracy for complex questions, sensitivity to prompt design, and challenges with interpretability. Conclusion. Thirty-one studies reported the use of GenAI in LBP management, mainly supporting diagnosis and treatment planning, patient education, research, and clinical documentation. Despite authors’ claims about promising uses of GenAI, it typically requires further refinement or review by human end-users. Critically, no studies addressed end-user experience, or clinical effectiveness, underscoring the need for robust clinical evaluation beyond the lab-based environment.

**Supplementary Information:**

The online version contains supplementary material available at 10.1007/s10916-026-02406-0.

## Introduction

Low back pain (LBP) is a highly prevalent condition that affects millions of people globally, leading to a significant socioeconomic burden and reduced quality of life [1]. Despite the availability of evidence-based guidelines for the management of LBP, there remains a significant gap between recommended practice and actual management of LBP. This includes overuse of low-value care (e.g., unnecessary rest, inappropriate use of diagnostic imaging) [2] and underuse of high-value care (e.g., individualised education and exercise programs) [3].

The advent of generative artificial intelligence (GenAI) presents an opportunity to address low-value care and increase high-value care for the management of LBP [4]. GenAI is a new type of artificial intelligence that began in 2017 [5]. Specifically, GenAI predicts the next word based on context or preceding text, enabling computers to follow prompts (user instructions to GenAI) to create conversations, reports, analyses, and potentially create clinical tools to automate healthcare processes [6]. Unlike other AI technologies that are designed for specific tasks or that rely on predefined content (e.g., robotic control systems and convolutional neural networks), GenAI is designed to generate new content on demand. ChatGPT is one example of GenAI developed by OpenAI [7]. Early research regarding the use of GenAI in healthcare reported potential benefits in the areas of ophthalmology and psychiatry for the detection of eye disease [8], providing treatment advice [9], simplifying radiology reports [10], producing high-quality clinical case summaries [11], and interpreting patient complaints [12]. However, preliminary research has highlighted critical deficiencies in accuracy (e.g., GenAI hallucination) [13], opacity (e.g., black-box algorithms) [14], and prejudice (e.g., reinforcement of bias) [15], alongside concerns regarding data privacy [13].

Therefore, the application of GenAI in the routine management of LBP should be conducted with caution, as the potential risks and benefits have not yet been rigorously evaluated [13]. Given the rapid development of GenAI, there is a need to summarise up-to-date evidence to map current usage and investigate existing gaps. To date, no scoping review on GenAI has comprehensively mapped the literature about its use in the management of LBP.

This study aimed to map and synthesise existing literature on how GenAI is used in the management of LBP, and in doing so, identify research needs. The main research question for this review was: How is GenAI being used for clinicians, patients, and decision-makers in the management of low back pain?

## Method

We conducted the scoping review following guidance for the conduct of the Scoping Reviews published by the Joanna Briggs Institute (JBI) [16]. The reporting guideline and checklist, Preferred Reporting Items for Scoping Review (PRISMA-ScR) [17], was used to guide the reporting of the review findings. The review protocol was registered on the Open Science Framework (OSF) [18].

### Inclusion/Exclusion Criteria

The Population, Concept, and Context Framework (PCC), as recommended by JBI [16], was used to formulate our inclusion and exclusion criteria in the selection of studies. To be included in this review, studies must have reported on GenAI in the field of the management of LBP (Table 1).

**Table 1:** Inclusion and exclusion criteria

Criteria Type	Details
Inclusion criteria	1. Studies that contained details about the management of LBP (e.g., diagnosis, imaging, and treatment.)
	2. Studies that contained details about using GenAI (e.g., chatbots such as ChatGPT)
	3. Studies that were published from January 1st, 2012, to July 8th, 2025.
	4. Research articles, including preprints (e.g., qualitative, quantitative, and mixed methods study designs), reviews, and case studies.
Exclusion Criteria	1. Studies written in a non-English language where translation was not available.
	2. AI techniques that were not GenAI (e.g., robotic control systems, single convolutional Neural Networks or traditional computer-aided diagnosis).

*GenAI *generative artificial intelligence

### Population

The population in this scoping review included patients, clinicians, and decision-makers who sought care for, or managed LBP, respectively. Consistent with standard diagnostic triage frameworks [1], this review included both non-specific LBP and specific spinal pathologies (e.g., radicular syndrome, malignancy). The management of LBP, as defined in this review, comprised all stages of management, including triage, clinical history-taking, examination, diagnostic imaging, treatment, appointment scheduling, clinical follow-up, and preventive advice.

### Concept

For the purposes of this review, GenAI in the management of LBP refers to AI systems that create new content on demand using natural language processing, and are capable of understanding, learning, and performing a wide array of tasks across various healthcare settings in the process of managing LBP [15]. For example, GenAI chatbots could be used as decision aids [19], potentially improving clinical workflow and empowering patients [4]. This contrasts with non-GenAI systems, which focus on specific tasks based on a preset template, rather than generating new content. Non-GenAI technologies include traditional computer-aided diagnosis, single convolutional Neural Networks, robotic control systems, clustering, factor analysis, and scoring systems.

To capture all potential examples of GenAI, we included research from January 1st, 2012, to July 8th, 2025. Hence, this review covered the period from 2012 to the most recent publication meeting our inclusion criteria.

### Context

Considering GenAI’s potential to perform a range of tasks, this study covered settings operationally defined as ‘clinical’ (involving real patients or clinical records, across all care settings), and ‘non-clinical’ (benchmarking via clinical vignettes, synthetic data, or common inquiries without patient interaction).

### Search Strategy

The search strategy was developed with the support of an academic librarian and covered peer-reviewed literature published after January 2012 (approximately five years prior to the advent of GenAI), using the following academic databases: The Cochrane Library, Medline, Cumulative Index to Nursing and Allied Health Literature (CINAHL), Scopus, Web of Science, and Embase. In addition, we searched preprint servers: Web of Science Preprint Citation Index, medRxiv, arXiv, and ProQuest. The search was conducted on July 8th, 2025. The search strategy (Ovid-Medline) is provided in the supplementary material (Appendix 1).

### Study Selection

Covidence software was used for literature management [20]. Title/abstract screening, followed by full-text screening, was performed independently by three reviewers (RT, IS, JC). Pilot testing of applying the inclusion criteria was conducted on 25 randomly selected abstracts prior to full screening. A senior researcher resolved conflicts if required.

### Data Extraction and Synthesis

A data extraction template (Excel spreadsheet, Supplementary Material 1) was piloted by three authors (RT, IS, JC) who independently extracted data from two included studies, chosen at random. Descriptive data extracted from each study were: author, year of publication, country of origin, title, study aims, methodology (including study design, setting, population, GenAI model/s, comparator), results, and author conclusions.

The included studies were categorised according to the primary purpose of GenAI implementation using a hybrid approach. Initial broad themes (specifically diagnosis, patient education, and user experience) were adapted from the framework by Kolding et al. [12], while additional sub-categories and prompt types were identified during the review process to capture the nuances of the LBP studies (Table 2).

**Table 2:** Categorization of included studies

Category	Primary user/perspective	Subcategory	Description
1. GenAI used for Diagnosis and Treatment	Clinicians	Answering specific questions about LBP	Studies that evaluated GenAI’s ability to answer specific questions related to diagnosis and treatment of LBP.
		Generating diagnosis and treatment advice from clinical cases	Studies that evaluated GenAI’s ability to generate diagnosis and treatment advice, based on a clinical scenario or electronic health records.
		Generating diagnosis and treatment advice from diagnostic imaging	Studies that evaluated GenAI’s ability to generate diagnosis and treatment advice, based on imaging reports or scans.
2. GenAI used for patient education and questions	Patients		Studies that evaluated GenAI’s ability to provide education materials or self-management plan for users with LBP.
3. Research support	Researchers		Studies that evaluated GenAI’s ability to support LBP research, such as perform evidence retrieval and article summaries.
4. Clinical documentation support	Clinicians		Studies that evaluated GenAI’s ability to assist with clinical documentation tasks, such as transforming unstructured imaging annotations.
5. Evaluation of user experience	Clinicians and Patients		Studies that explored how clinicians and patients perceive and interact with GenAI applications in managing LBP, such as behavioural intention, and expectation regarding its use.
6. Governance and policy	Healthcare system		Institutional or national policies to address common risks of GenAI use in LBP management, such as ethical implications, and safety concerns.

### Protocol Registration and Deviations

The protocol was registered in OSF (10.17605/OSF.IO/YX76D). Two deviations from the registered protocol occurred. First, protocol inclusion criterion #3 (studies conducted in any healthcare setting) was expanded to include studies that tested the interaction with GenAI using case-vignettes typically encountered in healthcare settings. This change facilitated a more comprehensive review of GenAI literature targeted for use in health care, which we believe better aligns with the purpose of our scoping review. Second, regarding measured variables listed in the protocol (e.g., barriers/facilitators), these were synthesised narratively within the "Results and Discussion" sections rather than reported separately. This allowed for a more integrated interpretation of the included studies.

## Results

### Search Results

**Fig. 1:**
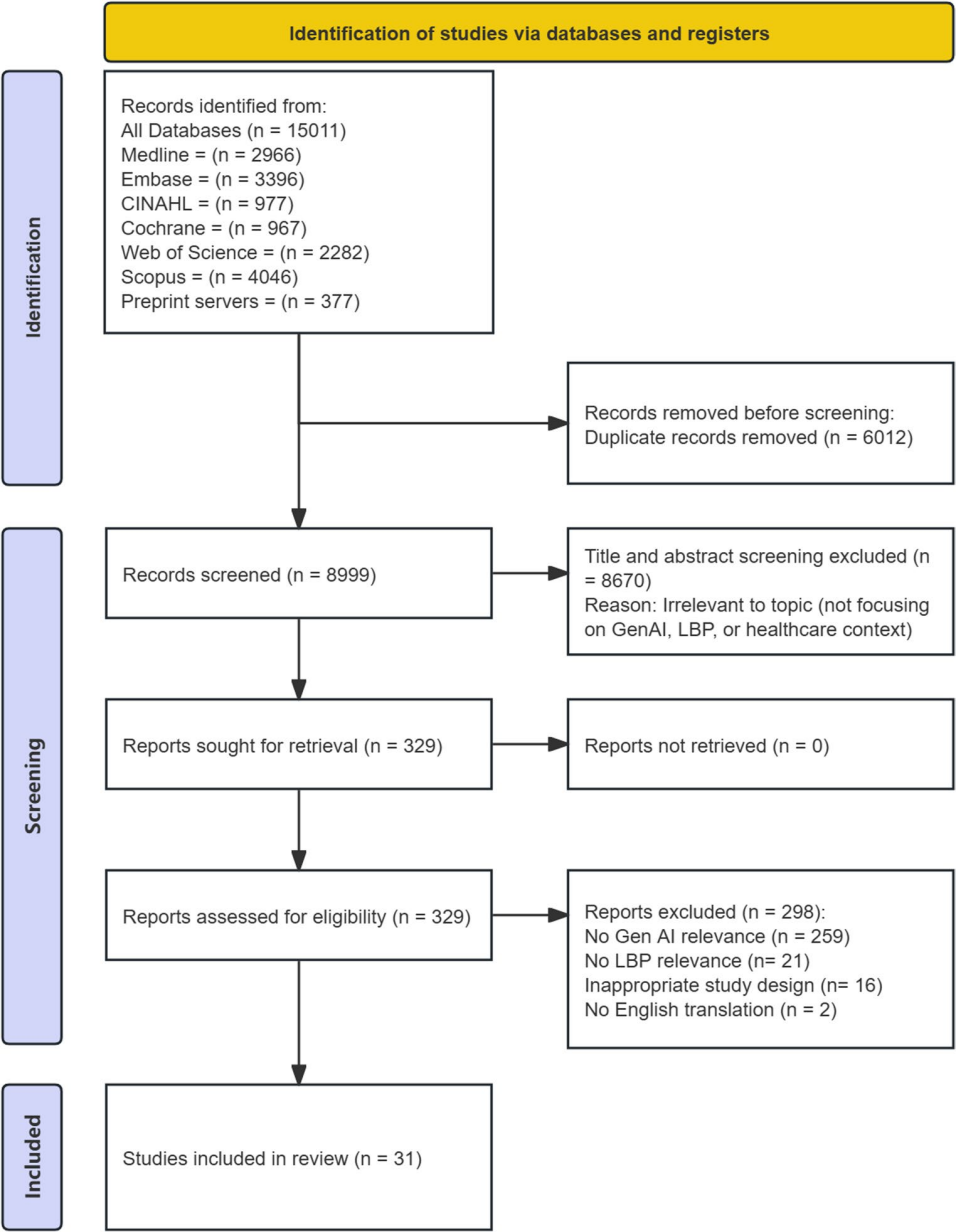
PRISMA flow chart for study inclusion

The search strategy identified 8,999 unique citations across six bibliographic databases and four preprint servers. After screening the titles/abstracts, 329 citations related to GenAI were eligible for full-text screening. Among these citations, 31 studies met our inclusion criteria (Fig. 1).

### Characteristics of Included Studies

Characteristics of the 31 included studies are provided in Table 3 (with detailed quantitative performance metrics available in Supplementary Table S1), and a summary of important findings across these categories is provided in Table 4. All studies were published in 2023 or later, with 15 studies from the USA [21–35], four from Europe [36–39], four from China [40–43], four from Turkey [44–47], and one each from Lebanon [48], Japan [49], Brazil [50], and South Korea [51]. Twenty studies were conducted in non-clinical settings (i.e., without interaction with patients or clinical records), nine studies were conducted in secondary care, and two in primary care settings. The most frequently used GenAI models were different versions of ChatGPT, featured in 30 (97%) studies, such as ChatGPT 3.5, 4, or a fine-tuned version of ChatGPT 4 (task-specific). Nine of the included studies also explored other GenAI models, including Google Bard [47, 50], Zephyr [39], Llama 3 Instruct [31, 35, 39], ERNIE Bot [40], DeepSeek [40], Copilot [36], Bing [50], Gemini 1.5 Pro [42], and Claude 3 [22, 36, 42]. Seventeen studies evaluated GenAI’s ability to answer specific questions related to the diagnosis and treatment of LBP [21, 24, 29, 32, 33, 38, 41, 45], analyse whole clinical cases [40, 44, 48, 49] or imaging reports [22, 25, 30, 39, 43]. Eight studies explored GenAI’s use in patient education [27, 35, 36, 42, 46, 47, 50, 51]. Five studies explored GenAI as a research support tool [23, 26, 28, 31, 37], and one focused on clinical documentation support [34].

**Table 3:** Characteristics of included studies

Author Year Country	Aims	Setting & Population	Back Pain condition	GenAI Models	Findings
**Diagnosis & Treatment (Specific questions)**					
Ahmed 2024 USA	Evaluate GenAI concordance with NASS guidelines across 28 questions.	Non-clinical	Degenerative spondylolisthesis	ChatGPT 3.5, ChatGPT 4	ChatGPT 4 responses were broadly concordant with guidelines and outperformed ChatGPT 3.5. However, authors advised caution regarding consulting GenAI for clinical recommendations due to inaccuracies in surgical treatment and explaining the value of spine care.
Gianola 2024 Italy	Evaluate GenAI accuracy against NICE guidelines across 9 questions.	Non-clinical	Non-specific LBP & Sciatica	ChatGPT 3.5	ChatGPT 3.5 had poor internal consistency and accuracy when compared to guideline recommendations for diagnosis and treatment of lumbosacral radicular pain.
Kayastha 2024 USA	Evaluate GenAI accuracy against NASS guidelines across 15 questions.	Non-clinical	Lumbar disc herniation with radiculopathy	ChatGPT 3.5, ChatGPT 4	ChatGPT 4 responses were broadly concordant with guidelines. However, authors also noticed the existence of GenAI hallucination and fabrication.
Lin 2025 Taiwan	Evaluate GenAI concordance with NASS guidelines across 31 questions.	Non-clinical	Spondylolisthesis	ChatGPT 3.5, ChatGPT 4	Both ChatGPT 4 and ChatGPT 3.5 responses were broadly concordant with guidelines but showed higher variance where guidelines lacked evidence, such as the topics of “Medical and intervention treatment” and “Value of spine care”.
Mejia 2024 USA	Evaluate GenAI accuracy against NASS guidelines across 29 questions.	Non-clinical	Lumbar disc herniation with radiculopathy	ChatGPT 3.5, ChatGPT 4	ChatGPT 4 responses were broadly concordant with guidelines and outperformed ChatGPT 3.5. Additionally, models were often able to provide useful information that was not included in the 2012 NASS guidelines.
Rajjoub 2024 USA	Evaluate GenAI accuracy against NASS guidelines across 16 questions.	Non-clinical	Degenerative lumbar stenosis	ChatGPT 3.5	ChatGPT 3.5 responses aligned with guideline recommendations and provided external literature-supported recommendations in areas of diagnosis and surgical intervention. However, some recommendations for pharmacological treatments were inaccurate, and the model occasionally fabricated evidence.
Safran 2025 Turkey	Evaluate GenAI performance against guidelines in physiotherapy assessment and rehabilitation across 20 questions.	Non-clinical	Lumbar disc herniation and Non-specific LBP	ChatGPT 4	ChatGPT 4 excelled in clarity, relevance, and accuracy compared against guidelines. However, the authors noted limitations in consistency and domain-specific reasoning, which necessitate human oversight and ongoing refinement.
Shrestha 2024 USA	Evaluate GenAI accuracy against NASS guidelines across 82 questions.	Non-clinical	Multiple specific causes, Non-specific LBP, Chronic LBP	ChatGPT 3.5	ChatGPT 3.5 responses were broadly concordant with guidelines, with performance improving after initial prompting. It also provided non-evidence-based advice in areas where the guidelines cited insufficient evidence.
**Diagnosis & Treatment (Clinical scenarios)**					
Chalhoub 2024 Lebanon	Evaluate GenAI accuracy against historical clinical records across 97 real cases.	Secondary care 97 patients with spinal pathologies	Spinal pathologies	ChatGPT 4	ChatGPT 4 demonstrated its utility in assisting with medical diagnoses. However, due to limitations such as reliance on outdated data and a failure to gather critical patient information, authors concluded that it cannot replace surgeons.
Chen 2025 China	Evaluate accuracy of fine-tuned GenAI (with external knowledge base) against spine clinicians’ advice across 80 LBP cases.	Primary care 80 chronic LBP patient complaint records	Chronic LBP	Fine-tuned ChatGPT 4, ChatGPT 4, ERNIE Bot, DeepSeek	Fine-tuned ChatGPT 4 achieved highest performance in accuracy, relevance, and clarity than other models when compared against clinicians’ advice, higher than other models.
Hirosawa 2023 Japan	Evaluate GenAI accuracy against clinicians’ advice across 3 created clinical cases.	Non-clinical	Musculoskeletal LBP & specific LBP	ChatGPT 3.5	ChatGPT 3.5 generated comprehensive differential diagnosis lists for common LBP complaints but was less accurate than clinicians.
Onan 2025 Turkey	Evaluate GenAI performance against orthopaedists decisions in developing exercise programs across 1 clinical case.	Secondary care 1 clinical case about lumbar disc herniation	Lumbar disc herniation	ChatGPT 4	ChatGPT 4 was able to generate realistic exercise programs for management of disc herniation. However, ChatGPT 4 recommendations did not fully address the complexities of real patient care and included overly demanding exercises.
**Diagnosis & Treatment (Imaging reports)**					
Almekkawi 2025 USA	Evaluate GenAI accuracy against spine surgeons in surgical decision-making and radiological detection across 5 MRI scans.	Secondary care 5 patient MRI scans of spine pathologies	Multiple spine pathologies	ChatGPT 4, Claude 3 Opus	ChatGPT 4 and Claude 3 Opus were able to provide detailed descriptions of MRI findings, including disc degeneration, herniations, and spinal canal stenosis, but with lower accuracy than experienced surgeons.
Khoylyan 2025 USA	Evaluate GenAI concordance with NASS guidelines in augmenting operative care decision-making across 17 clinical records.	Secondary care 17 clinical records including degeneration and spinal stenosis	Lumbar stenosis, Lumbar degeneration	ChatGPT 4	ChatGPT 4 aligned with NASS guidelines when evaluating indications for lumbar spine fusion surgery and was reported to be more adherent to guidelines than North American spine surgeons.
Moallem 2024 USA	Evaluate GenAI diagnostic accuracy against historical clinical records across 166 radiology reports.	Secondary care 166 patients; 50% with spondylolisthesis	Spondylolisthesis	ChatGPT 3.5	ChatGPT 3.5 identified spondylolisthesis with high accuracy when interpreting radiology reports.
Park 2024 UK	Evaluate GenAI performance in summarising and producing binary labels against original reports across 2281 MRI scans.	Secondary care 2286 MRI studies and 6844 Intervertebral Discs	Lumbar spinal stenosis	ChatGPT 4, Zephyr (7B),Llama 3 Instruct (8B)	All models achieved high labelling accuracy against original reports and outperformed traditional classifier.
Wang 2025 China	Evaluate GenAI multimodal performance (textual guideline adherence and MRI image interpretation) across 21 questions and 53 MRI scans.	Secondary care 53 patients; 31 with lumbar disc herniation	Lumbar disc herniation	ChatGPT 4o, ChatGPT 4o mini	Both models were effective in identifying lumbar disc herniation in MRI scans. However, responses were noted as "very difficult to read", and the occasional use of misleading terms (e.g., ‘tumour’) may increase patient anxiety.
**Patient education**					
André 2025 France	Evaluate GenAI alignment with HAS guidelines across 9 questions.	Non-clinical	Multiple types of LBP	ChatGPT 4, Copilot, Claude 3.5	GenAI models could serve as accessible educational resources for patients, providing guideline-based information and enhancing their understanding of LBP
Lieu 2025 USA	Evaluate GenAI accuracy and readability across 12 patient questions.	Non-clinical	Scoliosis	ChatGPT 3.5	ChatGPT 3.5 provided satisfactory responses as judged by clinicians. However, the estimated reading level (11th grade to college graduate) was deemed unrealistically high for the general patient population.
Liu 2024 China	Evaluate GenAI appropriateness with clinicians’ advice across 26 questions.	Non-clinical	Chronic LBP	ChatGPT 3.5, ChatGPT 4, Claude 3, Gemini 1.5 Pro	ChatGPT 4 and Gemini 1.5 Pro demonstrated superior appropriateness compared to other models in identifying risk factors and diagnosing chronic LBP, while remained less consistent regarding treatments and preventive measures.
Scaff 2024 Brazil	Evaluate GenAI performance against clinicians’ decisions across 30 patient questions.	Non-clinical	Multiple types of LBP	ChatGPT 3.5, Bing, Bard, ChatGPT 4	GenAI models generally provided moderately accurate but variable recommendations. All responses were classified as reasonably difficult to read. Notably, ChatGPT 4 provided safety-related information in 100% of its responses.
Yang 2024 Korea	Evaluate GenAI performance against clinicians’ advice in providing information to patients across 12 questions.	Non-clinical	Disc herniation	ChatGPT 4	ChatGPT 4 provided valid, safe, and useful information for patients seeking information on disc herniation.
Yilmaz(A) 2024 Turkey	Evaluate GenAI performance in providing preventative patient information against clinicians’ advice for one question.	Non-clinical	Musculoskeletal LBP	ChatGPT 3.5, ChatGPT 4	Both ChatGPT 3.5 and ChatGPT 4 contributed effectively to the prevention of LBP by delivering high-quality health information.
Yilmaz(B) 2024 Turkey	Evaluate GenAI accuracy in identifying red flags against guidelines across 70 questions.	Non-clinical	Symptoms of red flags	ChatGPT 3.5, Google Bard	Both models demonstrated strong performance in identifying LBP-related red flags from patient perspectives.
Zhao 2025 USA	Evaluate the accuracy of fine-tuned GenAI (with external knowledge bases) against clinicians’ decisions in developing patient education materials across 30 cases.	Non-clinical	Multiple types of LBP	ChatGPT 3.5 Turbo, ChatGPT 4, ChatGPT 4o, ChatGPT 4o mini, Llama 3-8b-Instruct (fine-tuned model with external knowledge bases)	Models generated accurate, personalised patient education materials, particularly when integrated with external knowledge bases. Notably, the fine-tuned model most consistently adhered to the requested 5th-grade reading level than other models.
**Research support**					
Anderson 2024 USA	Evaluate GenAI accuracy in extracting knowledge relationships across 116 chronic LBP research articles.	Non-clinical	Chronic LBP	ChatGPT 3.5	ChatGPT 3.5 demonstrated its capabilities in extracting knowledge relationships across different LBP areas, outperformed the traditional machine learning labelling approach.
Coraci 2023 Italy	Evaluate the validity of a GenAI-generated disability assessment against established tools across 20 patients.	Primary care 20 patients with a history of LBP	Non-specific LBP & Chronic LBP	ChatGPT 3.5	The GenAI-generated questionnaire demonstrated a strong correlation with established tools in Italian. However, it omitted items addressing social and sexual relationships, limiting its assessment of the quality of life.
Kurland 2025 USA	Evaluate GenAI summarization capability for complex neurosurgical questions.	Non-clinical	Neurosurgical spinal pathology	ChatGPT 4, ChatGPT 4o, ChatGPT 4 Turbo (Fine-tuned)	ChatGPT 4 Turbo achieved 97.5% citation accuracy, and ChatGPT 4o was the most cost-effective. However, all models tended to repeat citations rather than information synthesis.
Lotz 2023 USA	Evaluate GenAI accuracy in extracting knowledge relationships across 65 chronic LBP research articles.	Non-clinical	Chronic LBP	ChatGPT 3.5	ChatGPT 3.5 demonstrated its capabilities in extracting knowledge relationships across different LBP areas but also showed high inconsistency.
Nunes 2025 USA	Evaluate fine-tuned GenAI accuracy in predicting placebo responses from trial transcripts across 116 chronic LBP patients.	Secondary care Post-trial transcripts from 116 adults with chronic LBP	Chronic LBP	Fine-tuned Llama 3	GenAI-generated summaries of transcripts improved classification accuracy of the previous predictive model. Additionally, these summaries achieved a high perceived level of fit as judged by authors.
**Clinical documentation support**					
Yeasin 2024 USA	Evaluate GenAI performance in transforming semi-structured clinical notes into coherent paragraphs across 515 MRI reports.	Secondary care 515 MRI reports about lumbar spinal stenosis	Lumbar spinal stenosis	ChatGPT 4	ChatGPT 4 generated accurate and coherent lumbar spine radiology reports as judged by clinicians, although it did not achieve high diagnostic accuracy.

Abbreviations: *GenAI* generative artificial intelligence, *LBP* low back pain, *NICE* national institute for health and care excellence, *NASS* north american spine society, *HAS* haute autorité de santé, *MRI* magnetic resonance imaging

Performance metrics:

Accuracy/Concordance/Appropriateness: The proportion of agreement against a reference standard (guidelines or clinician advice)

Internal consistency: Similarity of GenAI responses across multiple rounds of question & answer

Diagnostic accuracy: The ability of a test to discriminate between the target condition and health

**Table 4:** Key findings across categories

Category	Studies (n)	Key findings
1. GenAI used for Diagnosis and Treatment	17	GenAI showed capability in generating clinically relevant advice. However, performance was inconsistent and heavily influenced by model versions and level of clinical detail stated in the prompt. Fabrication of evidence was identified as a significant limitation in multiple studies.
2. GenAI used for Patient Education and Questions	8	In most instances, GenAI generated accurate patient educational materials. However, two studies found the generated advice was often too complex or incomplete, potentially limiting patient comprehension or reducing engagement with the material.
3. Research Support	5	GenAI showed some potential in data extraction and generating clinical tools. However, manual verification of GenAI output is essential due to risks of mislabelling, miscategorising or omitting key terms.
4. Clinical Documentation Support	1	GenAI generated comprehensive imaging reports but was less accurate (single study) when compared to radiologists’ reports.

The categories represent the primary purpose of GenAI implementation within the included studies. Diagnosis and Treatment: Providing responses for clinical questions, clinical cases, or imaging

Patient Education and Questions: Providing patient-facing educational materials

Research Support: Assisting with LBP-related research activities

Clinical Documentation Support: Assisting with clinical documentation tasks

### GenAI Used for Diagnosis and Treatment

Seventeen studies evaluated GenAI as a potential support tool for generating diagnosis and treatment advice. These include answering specific questions about LBP (*n* = 8), generating diagnosis and treatment advice from clinical cases (*n* = 4), and generating diagnosis and treatment advice based on results from diagnostic imaging (*n* = 5).

#### Answering Specific Questions About Low Back Pain

Eight studies [21, 24, 29, 32, 33, 38, 41, 45] focused on the evaluation of GenAI’s ability to answer specific questions related to the diagnosis and treatment of LBP. Examples from this category include: “*Should devices (such as belts*,* corset*,* and/or foot orthotics) be used in the management of nonspecific low back pain and sciatica?*” (ChatGPT 3.5) [38]; and “*In adult patients*,* what is the relationship between the radiological grade of isthmic spondylolisthesis and expected clinical presentation?*” (ChatGPT 3.5/4) [41]. The most reported metric was ‘accuracy’ or ‘concordance’, defined here as the proportion of agreement against a reference standard (e.g., the North American Spine Society guidelines [52]). Reported performance of GenAI varied widely, depending on the GenAI model version [21, 24, 29, 41], with later versions having greater concordance (e.g., ChatGPT 4 > ChatGPT 3.5). In addition, when prompts were constructed with greater detail, alignment of the output to the reference standard improved [33]. However, authors also reported the variability and risks in GenAI that could hinder clinical implementation. For example, GenAI performance was reported to have higher variance (e.g., fabrication of evidence) when clinical practice guidelines contained conflicting or insufficient evidence related to the query [33, 41], or when asked about some types of management (e.g., lower concordance when recommending pharmacological management) [32]. Similarly, GenAI responses to specific questions included sporadic introduction of non-endorsed interventions for management of LBP (e.g., ChatGPT 4 recommended traction and electrotherapies) [38].

#### Generating Diagnosis and Treatment Advice from Clinical Cases

Four studies [40, 44, 48, 49] evaluated the ability of GenAI (ChatGPT 3.5, ChatGPT 4, and DeepSeek) to generate diagnosis and treatment plans based on clinical cases derived from electronic health records or expert-generated cases. The cases included a range of clinical details, requiring GenAI to integrate information and then generate advice. For example: “*Patient is a 72-year-old female associated with low back pain with irradiation to left lower limb*,* presence of night pain… you will give me the diagnosis and management plan…*”(ChatGPT 4) [48]. Authors indicated that supplementing GenAI with an external knowledge base enhanced its performance (e.g., ChatGPT 4 demonstrated higher accuracy for chronic LBP when supplemented) [40]. However, two studies found that GenAI responses were often of “low quality” and required clinician adjustments, such as prescribing overly demanding exercise plans for patients diagnosed with spinal pathology or disc herniation [44, 48].

#### Generating Diagnosis and Treatment Advice from Diagnostic Imaging

Five studies [22, 25, 30, 39, 43] evaluated the ability of GenAI (ChatGPT 3.5/4/4o/4o mini, Zephyr, Llama 3-instruct-8B, and Claude) to generate a diagnosis and surgical plan based on diagnostic imaging reports or scans. For example: “*You are a North American board-certified spine surgeon. You will determine the following clinical scenario should or should not be treated with a spinal fusion surgery? Image findings: Lumbar MRI L4-5 degenerative spondylolisthesis and severe bilateral later recess stenosis at L4-5…*” (ChatGPT 4) [25]. Across four studies [25, 30, 39, 43], GenAI was reported to be capable of interpreting both imaging reports and scans with high concordance, and generating detailed descriptions of the targeted spinal pathologies. Other reported advantages included greater adherence to guidelines compared to surveyed surgeons’ opinions [25], as well as potential for the local deployment of GenAI model (usually can preserve data locally, with lower cost compared to commercial versions of GenAI models) [39]. Errors in GenAI output were also reported. For example, one study reported that GenAI offered surgical treatment advice with only 20% accuracy [22]. Variation in performance was also attributable to prompt design, with different question styles producing concordance with clinical records ranging from moderate to very high [30].

### GenAI Used for Patient Education and Questions

Eight studies [27, 35, 36, 42, 46, 47, 50, 51] assessed the ability of GenAI (ChatGPT 3.5/4/4o/4o mini, Claude 3.5, and Llama 3-8b-Instruct) to provide educational information, such as self-management advice and medical knowledge directed at users with LBP. Examples from this category include: “*Should I exercise even if I have low back pain?*”(ChatGPT 4) [50]; and “*I have low back pain and unintentional weight loss*,* what shall I do?*” (ChatGPT 3.5/4) [46]. The educational material topics included advice such as prevention strategies [46], self-checks for potential red flag symptoms and risk factors [42, 47], exercise selection [51], cognitive behavioural interventions, and recommendations for use of nonsteroidal anti-inflammatory drugs [36]. Performance was reported to be greater for tasks such as disc herniation management, prevention strategies, and answering red flag related questions, compared with other scenarios. Additionally, one study reported that the GenAI model with external knowledge bases (e.g., specific medical databases or guidance) achieved higher accuracy than other basic models [35]. Several negative issues with the GenAI outputs were reported, such as producing content with overly complex vocabulary, and provision of incomplete advice. For example, two studies [27, 50] found that GenAI generated content at a college graduate reading level, which was difficult for patients to understand. Zhao et al. reported that although materials were successfully generated for fifth-grade reading level, ChatGPT 4 still produced incomplete statements such as: “*It’s important to understand how to take care of your back.*” without offering detailed clinical explanations [35].

### Research Support

Five studies [23, 26, 28, 31, 37] explored uses of GenAI in research-oriented tasks, such as identifying relationships across two domains of LBP, retrieving research articles, summarising interviews, or developing a disability assessment questionnaire.

Of these, two studies [23, 28] assessed the ability of ChatGPT 3.5 to identify relationships and summarise article findings across different subdomains of chronic LBP. For example: “*Given the following four categories and one biomedical paper abstract*,* classify the abstract as one of the four categories. Class 1: papers that only discussed biomechanical factors… Class 3: papers that discussed both psychological factors and biomechanical factors…*” (ChatGPT 3.5) [28]. In these two studies, authors reported that ChatGPT 3.5 demonstrated the ability to conduct tasks with over 60% accuracy, surpassing traditional approaches in performance but being less efficient [23], and containing errors such as mislabelling of relationships [28].

Two studies [26, 31] applied GenAI (fine-tuned Llama 3, fine-tuned ChatGPT 4/4o/4 turbo) to extract targeted information from large volumes of unstructured data, such as literature collections obtained via bibliometric methods and interview transcripts lasting up to 30 min. Reported strengths included higher efficiency, lower cost than traditional labour in extracting targeted information, and the ability to use the GenAI output to improve previous predictive models. However, issues with the complexity and readability challenges were also noted, leading to the use of a refinement prompt to eliminate redundancy and preserve all significant data [26].

One study used ChatGPT 3.5 to develop a disability assessment questionnaire for LBP in Italian [37]. The authors compared the final score completed by patients using the AI-generated questionnaire with those from other validated questionnaires (Oswestry Disability Index, Quebec Back Pain Disability Scale, Roland-Morris Disability Questionnaire). The authors reported that the AI-generated questionnaire showed strong correlations with other validated questionnaires but lacked key questions for assessing the quality of life (e.g., social and sexual relationships).

### Clinical Documentation Support

One study used ChatGPT 4 to support clinical documentation tasks such as generating structured reports from unstructured annotations [34]. ChatGPT 4 was reported to be able to generate coherent, grammatically structured reports from radiologists’ annotations of lumbar spine magnetic resonance imaging (MRI) scans, which previously contained many spelling and grammatical errors. However, the authors found that the model-generated diagnoses had only modest agreement with the original radiologists’ reports, with discrepancies that included mismatched or missing terms.

## Discussion

### Main Findings

This review synthesised the current use of GenAI in the management of LBP. A total of 31 studies met the inclusion criteria and were categorised based on the primary purpose of GenAI implementation. Most studies focused on different versions of ChatGPT.

Seventeen studies (55%) evaluated GenAI as a potential tool for generating diagnosis and treatment advice. While GenAI generally produced clinically relevant advice, accuracy varied markedly across studies and appeared to depend on both model capability and prompt design. Performance tended to improve with successive model iterations, richer prompts that specified clinical context and task requirements, and the use of external reference material such as published guidelines. However, the performance and utility of GenAI were limited by hallucinations, and response variability appeared to depend on the target condition. It was also unclear whether this response variability was influenced by the inherent limitations of the GenAI models themselves, differences in study methodology, or conflicting recommendations presented in guidelines [53].

Eight studies (26%) explored the role of GenAI in supporting users with LBP. GenAI was reported to provide educational materials with generally higher accuracy and less variability compared to the above seventeen studies. However, issues such as incomplete advice and readability challenges were noted, which could hinder user comprehension and understanding.

Five studies (16%) investigated GenAI applications in research, and one study evaluated its use in assisting with clinical documentation. GenAI was able to provide various forms of support, such as extracting literature and transforming unstructured data, often reported to perform better than traditional methods. Included studies suggested that human review would still be necessary, given that the GenAI generated responses could contain errors such as mislabelling or missing terms.

Regardless of specific implementation methods used, several recurring errors were frequently noted, such as inconsistency with evidence, model hallucinations (e.g., fabrication of evidence), reduced accuracy for complex clinical questions, sensitivity to prompt design, and readability challenges of outputs. Consequently, reported accuracy across tasks and authors’ perspectives regarding GenAI varied widely. These limitations were also reported as barriers to the safe and effective implementation of GenAI in future clinical practice.

Several included studies explored potential solutions to address these limitations, such as incorporating external knowledge bases, providing clinician adjustments, or designing better prompts to improve the accuracy and readability of GenAI responses. However, despite improvement in GenAI performance, the literature indicated the need for further refinement of GenAI and continual human oversight to maintain accuracy, accessibility and potentially patient safety.

### Compared to Other Studies

To our knowledge, this is the first scoping review to synthesise studies investigating how GenAI has been used across all aspects of managing LBP. As such, we can only compare our results with previous reviews of other healthcare conditions or established AI technologies (e.g., natural language processing). Consistent with findings in psychiatry, we observed current LBP GenAI literature was primarily conducted as prompt experiments to evaluate the quality of AI-generated content [12]. Regarding traditional natural language processing (non-generative), previous reviews indicated that the primary utility of AI-generated content lies in automating MRI report classification (e.g., identifying spinal stenosis), and mining large patient datasets for research [54]. These applications parallel the diagnosis and research support findings in this review. However, a greater body of evidence exists in psychiatry (more than 40 studies), featuring varied study designs such as surveys and interviews on user experience. Furthermore, studies in the natural language processing field frequently utilise massive datasets, often involving thousands of manually labelled reports for validation. In contrast, such a volume of evidence and range of methodologies were not observed in this scoping review on the LBP literature, despite our sensitive search strategy and broad inclusion criteria.

Compared to other clinician/patient support tools (e.g., non-GenAI mobile phone applications), we found less evidence supporting GenAI use in the management of LBP. For example, Self-Back [55], a non-GenAI decision support system for self-management of LBP, was developed through a well-defined research pathway that included preregistered protocols, qualitative studies, clinical trials, and follow-up evaluations. As application of GenAI in healthcare is an emerging field, there is an opportunity to adopt and adapt similar mature frameworks to guide future study designs. For example, GenAI responses based on detailed clinical information may present opportunities for tailored, person-centred healthcare solutions beyond those recommended in clinical practice guidelines [56].

Although we used a comprehensive search and applied broad inclusion criteria, we did not identify studies that evaluated user experience or assessed the effectiveness and cost-effectiveness of GenAI implementation. User experience, particularly perceptions of acceptability, trust, and risk, is a critical determinant of technology adoption [57]. As clinicians act as gatekeepers in LBP management, their specific perception of these factors will directly affect the feasibility of potential GenAI integration. Furthermore, it remains unknown how patient interactions with GenAI might impact the clinician-patient relationship, which also plays a vital role in therapeutic engagement and effectiveness [58]. Moreover, there is limited clarity on how GenAI should be applied to address gaps in LBP management, such as better clinical tools to be integrated within healthcare systems [59]. Effectiveness and cost-effectiveness are also central to evaluating the practical value of new technologies [60]. However, most studies focused on evaluating the quality of GenAI outputs, rather than testing their real-world impact on clinical decisions, patient outcomes, or cost-effectiveness in studies involving real clinicians or patients.

### Strength and Limitations of Review

We mapped GenAI usage across different healthcare settings and LBP conditions commonly encountered in primary, secondary and emergency healthcare. Key strengths of this scoping review included the use of a published protocol [18], and comprehensive coverage of both bibliographic databases and preprint servers. We included a broad definition of GenAI to capture all available evidence, and we conducted pilot testing before each stage of the scoping review to maintain consistency during screening and data extraction.

Limitations of this review were the variation in reference standards and evaluation methods across the included diagnosis and treatment studies, which hindered meaningful comparison of GenAI performance across studies. To assist with interpretation across studies, we harmonised terminology where applicable (e.g., studies used a range of terms to denote level of agreement with the reference standard: accuracy, concordance, or acceptance) and reported these differences descriptively in the "Results" section. Another limitation was the unclear definition of target users and application contexts. These issues sometimes led to overlapping scenarios (e.g., mixing patient and clinician-focused questions). To address this, we synthesised the evidence by grouping studies based on their primary purpose to better present the categorisation of included studies. Nevertheless, we advise caution when interpreting GenAI performance in aggregate given the variation in study designs.

Our review did not identify specific evidence regarding policies for GenAI in LBP management. This absence is likely because policy documents typically address broader aspects of healthcare, rather than focusing on specific conditions like LBP [61]. Consequently, our inclusion criteria may have excluded these broader governance and policy frameworks. While included studies highlighted implementation risks such as data privacy [38, 39, 46] and ethical standards [38, 50], addressing these issues likely requires clearly articulated policy frameworks, rather than piecemeal, study-level solutions, which are beyond the scope of this review.

### Implications for Clinicians, Researchers, and Decision Makers

The field of GenAI is rapidly evolving, presenting both new opportunities and distinct challenges for the management of LBP. Our findings carry important implications for clinicians, researchers, and decision-makers regarding the use of GenAI in LBP management.

For clinicians, potential benefits of GenAI included its accessibility, interpretability and ability to retrieve relevant information across different clinical tasks. GenAI capacity for rapid response and tailored advice may support clinical decision-making and patient education in managing LBP [62]. However, the practical utility of GenAI will also be limited by implementation challenges and complexities [63], such as variability in response quality (e.g., requiring clinician verification), additional workforce training, economic factors (e.g., subscription costs), and health equity and accessibility (e.g., for non-English speakers). Therefore, LBP management should not be delegated solely to GenAI tools and must remain under clinician supervision in clinical practice.

For researchers, studies reported higher performance in evidence retrieval, clinical interview analysis and summary of article findings, compared to traditional approaches. However, the use of inconsistent evaluation frameworks and the focus on lab-based evaluation of GenAI responses highlights the need for well-designed real-world studies involving end-users.

For decision-makers, governance frameworks that set clear requirements for data privacy and define accountability for GenAI-assisted decisions could help address these common risks [64]. These policies should support the safe integration of GenAI into LBP management [61]. Besides, clinical pathways and other standardised tools for managing LBP may be redesigned to integrate GenAI, but only after its clinical effectiveness and cost-effectiveness have been rigorously established [65].

### Unanswered Questions and Future Research Recommendations

Despite the possibility that GenAI may already have been used informally by patients for many healthcare related tasks [13, 14, 66], key evidence regarding the effectiveness and cost-effectiveness of GenAI remains unanswered. Most included studies focused on evaluating the quality of GenAI outputs, but few examined its effect on clinical decision-making, patient outcomes, or the value of care. In addition, the limited evaluation of user experience in the included studies contributes to an incomplete picture of GenAI’s role in LBP management. Finally, systemic risks such as the reinforcement of societal biases (e.g., GenAI training and testing in predominantly English-language based environments) and copyright integrity (e.g., duplicate protected material) remain largely unexplored in the current LBP literature.

To advance the field of GenAI in LBP management, we propose the following directions for future research. First, to enhance clinical relevance and clarity of findings, we recommend future studies apply evaluation frameworks when assessing GenAI in the LBP management (e.g., Saragiotto et al. evaluated GenAI using question derived from patient common inquiries and applied validated evaluation framework [50]). Second, we encourage the field to move beyond basic knowledge testing and isolated prompt experiments to instead address diverse clinical needs involving real-world users. With clearly articulated policy frameworks, the role of GenAI in LBP management could be more comprehensively understood.

## Conclusion

Our review identified a range of GenAI usage in the management of LBP for supporting clinicians, patients, and researchers. These uses included assistance with making diagnosis and treatment plans, providing patient educational materials, supporting research and clinical documentation. Most studies focused on different versions of ChatGPT and reported that GenAI models demonstrated variable accuracy, indicating that GenAI still requires further refinement or human review for end users. No studies evaluated user experience or assessed effectiveness and cost-effectiveness of implementing GenAI in this field. Future studies should apply robust evaluation frameworks and address diverse clinical needs involving real-world users to better understand the role of GenAI in LBP management.

## Supplementary Information

Below is the link to the electronic supplementary material.

Supplementary Material 1

Supplementary Material 2

Supplementary Material 3

## Data Availability

No datasets were generated or analysed during the current study.
