# Supplementary material for: Use of Generative Artificial Intelligence in the Management of Low Back Pain: a Scoping Review
Source: J Med Syst. 2026 May 9;50(1):75. doi: 10.1007/s10916-026-02406-0 (PMC13157360; doi:10.1007/s10916-026-02406-0)
Supplement: Supplementary file 2 — Supplementary Material 2 [file 10916_2026_2406_MOESM2_ESM.docx]

# Supplementary materials

### Quantitative performance metrics for GenAI

| Table S1. Detailed quantitative performance metrics of the included studies | | | | | | |
| --- | --- | --- | --- | --- | --- | --- |
| Author Year Country | Aims | Setting & Population | Back Pain conditions | GenAI Models | Method | Result |
| **Diagnosis &Treatment (Specific questions)** | | | | | | |
| Ahmed 2024  USA | To test the concordance of GenAI in the diagnosis and treatment of degenerative spondylolisthesis against recommendations from CPG | Non-clinical | Degenerative spondylolisthesis | ChatGPT 3.5, ChatGPT 4 | The study authors rated the concordance (%) of ChatGPT responses compared to CPG (2014 NASS) across 28 questions. The proportion of response that align with CPG (“concordance”), omitted key points (“insufficient”), and made unsupported claims (“over-conclusive”) were reported. | Compared to the CPG, ChatGPT 4 achieved:  -Concordance: 68% -Insufficient: 33% -Over-conclusive: 67% ChatGPT 3.5 performance scored: 46%, 20% and 80%, respectively. |
| Gianola 2024  Italy | To test the accuracy (proportion of agreement) of GenAI in the diagnosis and treatment of LBP against CPG | Non-clinical | Non-specific LBP & Sciatica | ChatGPT 3.5 | The study authors rated the accuracy (%) of ChatGPT responses compared to CPG (2016 NICE) across 9 questions. The proportion of response that align with CPG (“accuracy”), and internal consistency across three rounds of responses were reported. | Compared to the CPG, 33% of ChatGPT 3.5 response were accurate. The internal consistency across three rounds of responses was 49%. |
| Kayastha 2024  USA | To test the concordance of GenAI in the diagnosis and treatment of lumbar disc herniation with radiculopathy against CPG | Non-clinical | Lumbar disc herniation with radiculopathy | ChatGPT 3.5, ChatGPT 4 | The study authors rated the accuracy (%) of ChatGPT responses compared to CPG (2012 NASS) across 15 questions. The proportion of response that align with CPG (“accuracy”), included supplementary advice beyond CPG (“supplementary advice”), made unsupported claims (“over-conclusiveness”), or omitted important details (“incomplete”) were reported. | Compared to the CPG, ChatGPT 4 achieved:  -Accuracy: 67%. -Supplementary advice: 67%  -Over-conclusiveness: 33%  -Incomplete: 40% ChatGPT 3.5 performance scored: 47%, 100%, 47% and 40%, respectively. |
| Lin  2025 Taiwan | To test the concordance of GenAI models on spondylolisthesis against CPG | Non-clinical | Spondylolisthesis | ChatGPT 3.5, ChatGPT 4 | The study authors rated the concordance (%) of ChatGPT responses against CPG (2014 NASS) across 31 questions. Concordance with NASS guidelines were reported. | Compared to the CPG ChatGPT 4 achieved:  -General concordance: 42% -Category-Specific Performance: 46% -Clear vs. Ambiguous Guidelines: 91% ChatGPT 3.5 performance scored: 45%, 54% and 82%, respectively. |
| Mejia  2024  USA | To test the concordance of GenAI in the diagnosis and treatment of disc herniation with radiculopathy against CPG | Non-clinical | Lumbar disc herniation with radiculopathy | ChatGPT 3.5, ChatGPT 4 | The study authors rated the concordance (%) of ChatGPT responses compared to CPG (2012 NASS) across 29 clinical scenarios. The proportion of response that aligned with CPG (“accuracy”), included supplementary advice beyond CPG (“supplementary advice”), made unsupported claims (“over-conclusiveness”), or omitted important details (“incomplete”) were reported. | Compared to recommendations from CPGs, ChatGPT 4 achieved:  -Accuracy: 59% -Supplementary advice: 93% -Over-conclusiveness: 45% -Incomplete: 28% ChatGPT 3.5 performance scored: 52%, 83%, 48% and 38%, respectively. |
| Rajjoub 2024 USA | To test the accuracy (proportion of agreement) of GenAI in the diagnosis and treatment of degenerative lumbar stenosis against CPG | Non-clinical | Degenerative lumbar stenosis | ChatGPT 3.5 | The study authors rated the accuracy of ChatGPT responses compared to CPG (2012 NASS) and systematic review evidence across 16 questions. The precision of responses aligned with CPGs and the ability to provide relevant advice beyond guidelines were described qualitatively. | Compared to CPG and review evidence, ChatGPT 3.5 responses were reported similar to findings in the current literature in areas of definition, history, diagnostic test and surgical intervention (excluding non-surgical ones), and provided external literature-supported recommendations in physical exercise and therapy, manipulation, ancillary treatments (excluding pharmacological treatments and physical findings). |
| Safran  2025 Turkey | To test the performance of GenAI models in physiotherapy assessment and rehabilitation for LBP against CPG | Non-clinical | Lumbar disc herniation and Non-specific LBP | ChatGPT 4 | The study authors rated the performance (5 points) of ChatGPT responses against clinician’s advice across 20 questions. The relevance, accuracy, clarity, completeness and consistency of response were reported. | Compared to clinician’s advice, ChatGPT 4 achieved:  -Relevance: 4.0-4.5/5 -Accuracy: 5/5 -Clarity: 4.5-5/5 -Completeness: 3.5-5/5 -Consistency: 3-4/5 |
| Shrestha 2024 USA | To test the accuracy (proportion of agreement) of GenAI in the diagnosis and treatment of LBP against CPG | Non-clinical | Multiple specific causes & Non-specific LBP & Chronic LBP | ChatGPT 3.5 | The study authors rated accuracy (%) of ChatGPT responses compared to CPG (2020 NASS) and review evidence across 82 questions. The proportion of response that align with CPG and review evidence (compared at evidence level), performance before and after expert-framing prompt were reported. | Compared to CPG and review evidence, ChatGPT 3.5 achieved:  -Guidelines with recommendations:  65% before prompted and 72% after prompted -Guidelines with insufficient/conflicting evidence:  46% before prompted and 58% after prompted -Guidelines with no studies available:  49% before prompted and 16% after prompted. |
| **Diagnosis &Treatment (Clinical scenarios)** | | | | | | |
| Chalhoub  2024 Lebanon | To test the accuracy (proportion of agreement) of GenAI in the diagnosis and treatment of spinal pathologies against historical clinical records | Secondary care   97 patients with spinal pathologies | Spinal pathologies | ChatGPT 4 | The study authors rated the accuracy (%) of ChatGPT 4 responses with historical clinical records across 97 real cases. The accuracy of correct diagnosis, and treatment effectiveness were reported. | Compared to historical clinical records, 70% diagnosis of ChatGPT 4 responses were accurate, 95% of treatment options were suitable. |
| Chen  2025 China | To test the accuracy of GenAI models with external knowledge base for the diagnosis and treatment of chronic LBP against spine clinicians’ advice. | Primary care  80 chronic LBP patient complaint records. | Chronic LBP | Fine-tuned ChatGPT 4, ChatGPT 4, ERNIE Bot, DeepSeek | The study authors collected latest research papers and medical guidelines to build a knowledge base for fine-tuned ChatGPT 4. Then rated the accuracy of GenAI model responses against clinicians’ decision across 80 patient complaint records about chronic LBP. Accuracy, relevance, clarity, benefit, completeness of responses was reported | Compared to clinicians’ decision, fine-tuned ChatGPT 4 achieved: Accuracy: 4.4/5 Relevance: 4.4/5 Clarity: 4.4/5 Benefit: 4.4/5 Completeness: 4.4/5 Overall, it received an excellent total score of 4.4, further verifying its leading position in generative models. |
| Hirosawa 2023 Japan | To test the accuracy (proportion of agreement) of GenAI in the diagnosis of LBP against clinicians’ advice | Non-clinical | Musculoskeletal LBP & specific LBP | ChatGPT 3.5 | The study authors rated accuracy (%) of ChatGPT responses with clinicians’ advice across 3 created clinical cases (Remaining 12 cases were unrelated). Proportion of response made correct diagnosis compared to given answer (Accuracy) was reported. | Compared to clinicians’ advice, 66.7% of ChatGPT 3.5 responses were accurate, inferior to another group of clinicians’ accuracy (83%) in all 15 questions. |
| Onan  2025 Turkey | To evaluate the performance of GenAI in developing tailored 8-week exercise programs for patients with lumbar disc herniation against orthopaedist’s decision | Secondary care  1 clinical case about lumbar disc herniation | Lumbar disc herniation | ChatGPT 4 | The study authors rated the performance of GenAI responses against orthopaedist’s decision across 1 real clinical case. Evaluation of exercise programs was reported. | Compared to orthopaedist’s decision, ChatGPT 4 was able to generate exercise programs for commonly searched diagnoses; however, its recommendations did not fully address the complexities of real patient care and included overly demanding exercises. |
| **Diagnosis &Treatment (imaging reports)** | | | | | | |
| Almekkawi 2025 USA | To evaluate the accuracy of GenAI models in surgical decision-making and radiological detection for spine pathologies against spine surgeons’ decision. | Secondary care  5 Patient MRI images on spine pathologies | Multiple spine pathologies | ChatGPT 4, Claude 3 Opus | The study authors rated the accuracy of GenAI model responses against spine surgeons’ decision across 5 written clinical scenarios with MRI images about spine pathologies. Accuracy of decision-making, performance in detecting and describing pathologies on MRI images were reported. | Compared to surgeons’ decision, Both ChatGPT and Claude demonstrated an accuracy of 20% (1 of 5 cases), and both models provided detailed descriptions of the findings, including disc degeneration, herniations, and spinal canal stenosis. |
| Khoylyan  2025 USA | To evaluate the concordance of GenAI in augmenting operative care decision-making for lumbar stenosis or degeneration | Secondary care  17 clinical records including degeneration and spinal stenosis | Lumbar stenosis, Lumbar degeneration | ChatGPT 4 | The study authors rated the accuracy of GenAI model responses against 2023 NASS guidelines across 17 real clinical records and image report. Precision, recall, and F1 score of the GenAI responses, and the alignment with NASS guidelines were reported. | Compared to guidelines, ChatGPT 4 achieved a precision, recall, and F1 score of 90%. According to a survey about North American spine surgeons, ChatGPT 4 is more adherent to NASS guidelines when evaluating the indications for lumbar spine fusion surgery. |
| Moallem 2024 USA | To test the diagnostic accuracy of GenAI in the diagnosis of spondylolisthesis against historical clinical records | Secondary care  166 patients; 50% with spondylolisthesis | Spondylolisthesis | ChatGPT 3.5 | The study authors rated diagnostic accuracy of ChatGPT responses with radiology reports across 166 real cases. The sensitivity, specificity, precision, and accuracy of responses were reported. | Compared to the historical clinical records, ChatGPT 3.5 achieved 94% sensitivity, 98.8% specificity, 97.5% precision, and 96.4% accuracy. |
| Park  2024 UK | To evaluate the performance of GenAI models in summarising and producing binary labels from imaging reports about lumbar spinal stenosis | Secondary care  2286 MRI studies and 6844 Intervertebral Disc (part of reported related to lumbar spinal stenosis) | Lumbar spinal stenosis | ChatGPT 4, Zephyr (7B), Llama3 Instruct (8B) | The study authors asked the model to generate a summary of the report based on the target condition, and then using the summary to assign a binary label (Yes/no). Later the data was used to train a traditional classifier. Assessment of labelling accuracy, and the balance of accuracy and completeness (F1-score) were reported. | Compared to original reports, all models exceeded a balanced accuracy and F1-score of 0.9/1 in labelling, outperformed previous models (traditional classifier achieved balanced accuracy of 0.78/1). |
| Wang  2025 China | To evaluate clinical support capabilities of GenAI models in the diagnosis and readability for lumbar disc herniation against clinician’s decision | Secondary care  53 patient MRIs from the inpatient case database, 31 with lumbar disc herniation and the rest not | Lumbar disc herniation | ChatGPT 4o, ChatGPT 4o mini | The study authors rated accuracy of ChatGPT responses against 2012 NASS guidelines across 21 textual questions, and image recognition accuracy across 53 lumbar disc herniation MRIs. Accuracy, completeness, reliability, and readability levels were reported. | Compared to CPG, both models in textual questions achieved -Accuracy: 4.6/5 -Completeness: 5/5 -Reliability: 6/7 Both model responses were described as ‘‘very difficult to read’'.  Compared to clinician’s decision in imaging recognition. The model’s overall accuracy was 0.81, sensitivity was 0.87, and specificity was 0.7. |
| **Patient education** | | | | | | |
| André  2025 France | To evaluate the relevance of GenAI in treatment for common LBP against CPG | Non-clinical | Multiple types of LBP | ChatGPT 4, Copilot, Claude 3.5 | The study authors rated the accuracy of GenAI model responses against Haute Autorité de Santé guidelines across 9 questions. Consistency with CPG, and textual similarity among responses were reported. | Compared to CPG, ChatGPT and Claude each aligned with CPG in 5 out of 9 responses, while Copilot aligned in 4 out of 9 responses. Textual similarity among responses: ChatGPT 88.5%, Claude 88.3%, Copilot 90% |
| Lieu  2025 USA | To evaluate the performance of GenAI in answering patient questions about scoliosis against surgeon’s advice. | Non-clinical | Scoliosis | ChatGPT 3.5 | The study authors rated the performance of GenAI against surgeon’s decision across 12 questions about scoliosis.  Accuracy, clarity and readability level of the responses were reported. | Compared to surgeon’s decision, ChatGPT 3.5 achieved a score of 2.4 out of 4 for accuracy and clarity. The estimated reading level of its response ranged from 11th grade to college graduate. |
| Liu  2024 China | To test the appropriateness rate of GenAI in various stages of Chronic LBP against clinicians’ advice | Non-clinical | Chronic LBP | ChatGPT 3.5, ChatGPT 4, Claude 3, Gemini 1.5pro | The study authors rated the appropriateness (%) of ChatGPT responses against clinicians’ advice across 26 questions. The proportion of response that align with clinicians’ advice (“appropriateness”) was reported. | Compared to clinicians’ advice, ChatGPT 4 scored an appropriateness rate of 96.1%. Gemini scored 92.3%, while ChatGPT 3.5 and Claude 3 scored 80.7%. |
| Scaff  2024 Brazil | To evaluate the performance of GenAI in answering patient questions on self-management, risk factors, conservative and surgical treatment for LBP against clinician’s decision. | Non-clinical | Multiple types of LBP | ChatGPT 3.5, Bing, Bard, ChatGPT 4 | The study authors rated the performance of GenAI against surgeon’s decision across 30 questions about multiple types of LBP. Accuracy, readability level, and disclaimers about health advice of responses were reported. | Compared to clinician’s decision, 597 (55.8%) of the GenAI generated recommendations were classified as accurate, 451 (42.1%) as inaccurate and 21 (1.9%) as unclear. All responses were classified as reasonably difficult to read. ChatGPT 4 presented safety-related information in 100% of the responses generated. |
| Yang  2024 Korea | To test the performance of GenAI in the diagnosis and treatment of herniated lumbar disc vs clinicians’ advice | Non-clinical | Disc herniation | ChatGPT 4 | The study authors rated the performance (rated on a 0-4 scale) of ChatGPT 4 responses with clinicians’ advice across 12 questions. Proportions and response levels containing erroneous information (validity), content safety (safety), and perceived usefulness for patients (utility) were reported. | Compared to clinicians’ advice, 75% of ChatGPT responses were validated (4 points), 92% were safe, 75% were useful. |
| Yilmaz(A) 2024 Turkey | To test the quality of GenAI in the diagnosis and treatment of musculoskeletal LBP vs clinicians’ advice | Non-clinical | Musculoskeletal LBP | ChatGPT 3.5, ChatGPT 4 | The study authors rated the quality (%) of ChatGPT responses with clinicians’ advice in 1 question (Remaining 14 cases were unrelated). Completeness of response, lack of false information, supporting evidence, appropriateness and relevance of response were assessed. However, only the overall performance regarding LBP was reported. | Compared to clinician’s advice, ChatGPT 3.5 overall score achieved 4.8, same with ChatGPT 4. Regarding all 15 questions, ChatGPT 3.5 received average scores between 3.7 and 4.2 across different metrics, while ChatGPT 4 received scores ranging from 3.8 to 4.6. |
| Yilmaz(B) 2024 Turkey | To test the accuracy (proportion of agreement) of GenAI in the diagnosis and treatment of LBP red flags vs CPGs | Non-clinical | Symptoms of red flags | ChatGPT 3.5, Google Bard | The study authors rated accuracy (5 points) of ChatGPT 3.5 responses with CPGs across 70 questions (single or multiple symptoms). Proportion of accurate information in the responses were reported. | Compared to CPG and review evidence, ChatGPT 3.5 achieved: -58 Single symptoms: ranging from 3.5 to 3.9/5 -12 Multiple symptoms: ranging from 4.0 to 4.3/5 Google Bard scored: -58 Single symptoms: ranging from 3.4 to 3.8/5 -Multiple symptoms: ranging from 3.5 to 3.7/5. |
| Zhao  2025 USA | To evaluate the performance of GenAI with external knowledge base in developing patient education materials for LBP against clinician’s decision. | Non-clinical | Multiple types of LBP | ChatGPT 3.5 Turbo, ChatGPT 4, ChatGPT 4o, ChatGPT 4o mini, Llama-3-8b-Instruct (external knowledge base) | The study authors rated the performance of GenAI with external knowledge base in generating patient education materials across 30 patient cases that were generated by ChatGPT 4o. Redundancy, accuracy, completeness, and readability level of responses were reported. | Compared to clinician’s decision, Llama 3 with external knowledge base achieved highest redundancy (4.3/5) and accuracy score (3.5/5), and most consistently in producing content at a 5th-grade reading level as requested. Only inferior to ChatGPT 4o in completeness (3.6/5). |
| **Research support** | | | | | | |
| Anderson 2024  USA | To test accuracy (proportion of agreement) of GenAI models in identifying relationships across two Chronic LBP areas. | Non-clinical | Chronic LBP | ChatGPT 3.5 | The study authors used a ChatGPT-based tool to map relationships across 116 Chronic LBP studies on cognitive therapy and altered gait pattern. Precision and recall of labelling, and the balance of accuracy and completeness (F1-score) of relationship identification were reported. | Compared to assessment by the research team, ChatGPT 3.5 identified relationships with 57% precision, 74% recall and 70% F1-score, overpassed the traditional ontology-based machine labelling approach. |
| Coraci 2023 Italy | To compare the effectiveness of questionnaire generated by GenAI in the disability assessment of LBP vs other validated LBP assessment questionnaire. | Primary care  20 patients with a history of LBP | Nonspecific LBP & Chronic LBP | ChatGPT 3.5 | The study author rated effectiveness of a 10-item Italian LBP assessment questionnaire generated by ChatGPT (Chat-Q) against other validated questionnaires (Oswestry disability index/Quebec back pain disability scale/Roland-Morris Disability Questionnaire). Comparison on final scores and explored domains of questionnaires were reported. | Compared to other questionnaires, the Chat-Q reported as a strong correlation with the ODI and a moderate correlation with QBPDS. However, the absence of items addressing social and sexual relationships relevant to quality of life was noted as limitations. |
| Kurland 2025 USA | To evaluate the summarization capability of GenAI models in selecting relevant evidence to answer complex neurosurgical questions for LBP | Non-clinical | Neurosurgical spinal pathology | ChatGPT 4, ChatGPT 4o,  ChatGPT 4 Turbo (fine-tuned) | The study authors implemented a ChatGPT-based summarisation algorithm, including automatically extract relevant articles, refine response, and revise structure. Time and cost of AI-generated summaries, accuracy of summaries and associated Citations were reported. | Compared with original ChatGPT 4, the fine-tuned ChatGPT model response was more focused, robust and contained more details. Fine-tuned GPT 4 was the superior LLM with a 97.5% citation accuracy (n =202).   ChatGPT 4o was the cheapest model, costing <$0.50 to generate each summary. ChatGPT 4 costed $2.50.   All the model tended to repeat citations instead of combining or synthesizing points across the same article. |
| Lotz  2023 USA | To test the accuracy (proportion of agreement) of GenAI models in identifying relationships across two Chronic LBP areas. | Non-clinical | Chronic LBP | ChatGPT 3.5 | The study authors used a ChatGPT-based tool to extract research focus and map relationships across 65 Chronic LBP studies on psychological factors and biomechanical mechanisms areas. The accuracy of labelling and the F1-score for relationship identification were reported | Compared to assessment by the research team, ChatGPT 3.5 extracted research focus and identified relationships across studies with 60% accuracy. F1-score ranged from 36% to 70%. |
| Nunes  2025 USA | To evaluate whether a fine-tuned GenAI model improves the prediction and interpretation of placebo responses in chronic LBP RCTs | Secondary care    Post-trial transcripts from 116 adults with chronic LBP in two naproxen–placebo RCTs | Chronic LBP | Fine-tuned Llama-3 | The study authors rated performance of a fine-tuned context-sensitive Llama-3 model on chronic-pain texts to classify placebo responders and uncover latent semantic topics linked to placebo response. Classification accuracy and improvement over the previous predictive model were reported | Compared to original reports, Llama-3 achieved a classification accuracy of 74%, and the Llama-3 response improved the accuracy of previous predictive model from 67% to 74%. |
| **Clinician documentation support** | | | | | | |
| Yeasin 2024 USA | To evaluate the performance of GenAI models in transforming semi-structured clinical notes into more coherent paragraph structures based on MRI reports about lumbar spinal stenosis | Secondary care 515 MRI reports about lumbar spinal stenosis | Lumbar spinal stenosis | ChatGPT 4 | The study authors entered 515 reports about lumbar spinal stenosis to ChatGPT 4, with additional instructions to reformulate the assessment in the report into structured paragraphs. Diagnostic completeness, novel diagnostic detection, and diagnostic correspondence of ChatGPT response were reported. | Compared to original reports, ChatGPT 4 captured 24.7% of the original diagnostic information, and introduced new diagnostic content in 69.8% of reports. And 26.8% agreement between the generated reports and the original diagnoses from the radiologist’s reports. |

**Abbreviations**: GenAI: Generative Artificial Intelligence; LBP: Low Back Pain; NICE: National Institute for Health and Care Excellence; NASS: North American Spine Society; HAS: Haute Autorité de Santé; MRI: Magnetic Resonance Imaging.

**Performance metrics**:

Accuracy/Concordance/Appropriateness: The proportion of agreement against reference standards (guidelines or clinician advice).

Internal consistency: Similarity of GenAI responses across multiple rounds of question & answer.

Diagnostic accuracy: The ability of a test to discriminate between the target condition and health.

F1-score: The combined measure of precision and recall, reflecting a model's balanced predictive accuracy.

### Preferred Reporting Items for Systematic reviews and Meta-Analyses extension for Scoping Reviews (PRISMA-ScR) Checklist

| **SECTION** | **ITEM** | **PRISMA-ScR CHECKLIST ITEM** | **REPORTED ON PAGE #** |
| --- | --- | --- | --- |
| **TITLE** | | | |
| Title | 1 | Identify the report as a scoping review. | 1 |
| **ABSTRACT** | | | |
| Structured summary | 2 | Provide a structured summary that includes (as applicable): background, objectives, eligibility criteria, sources of evidence, charting methods, results, and conclusions that relate to the review questions and objectives. | 1-2 |
| **INTRODUCTION** | | | |
| Rationale | 3 | Describe the rationale for the review in the context of what is already known. Explain why the review questions/objectives lend themselves to a scoping review approach. | 2-3 |
| Objectives | 4 | Provide an explicit statement of the questions and objectives being addressed with reference to their key elements (e.g., population or participants, concepts, and context) or other relevant key elements used to conceptualize the review questions and/or objectives. | 2-3 |
| **METHODS** | | | |
| Protocol and registration | 5 | Indicate whether a review protocol exists; state if and where it can be accessed (e.g., a Web address); and if available, provide registration information, including the registration number. | 3-5 |
| Eligibility criteria | 6 | Specify characteristics of the sources of evidence used as eligibility criteria (e.g., years considered, language, and publication status), and provide a rationale. | 3-4 |
| Information sources* | 7 | Describe all information sources in the search (e.g., databases with dates of coverage and contact with authors to identify additional sources), as well as the date the most recent search was executed. | 4 |
| Search | 8 | Present the full electronic search strategy for at least 1 database, including any limits used, such that it could be repeated. | 4 |
| Selection of sources of evidence† | 9 | State the process for selecting sources of evidence (i.e., screening and eligibility) included in the scoping review. | 4-5 |
| Data charting process‡ | 10 | Describe the methods of charting data from the included sources of evidence (e.g., calibrated forms or forms that have been tested by the team before their use, and whether data charting was done independently or in duplicate) and any processes for obtaining and confirming data from investigators. | 4-5 |
| Data items | 11 | List and define all variables for which data were sought and any assumptions and simplifications made. | 4-5 |
| Critical appraisal of individual sources of evidence§ | 12 | If done, provide a rationale for conducting a critical appraisal of included sources of evidence; describe the methods used and how this information was used in any data synthesis (if appropriate). | N/A |
| Synthesis of results | 13 | Describe the methods of handling and summarizing the data that were charted. | 4-5 |
| **RESULTS** | | | |
| Selection of sources of evidence | 14 | Give numbers of sources of evidence screened, assessed for eligibility, and included in the review, with reasons for exclusions at each stage, ideally using a flow diagram. | 5 |
| Characteristics of sources of evidence | 15 | For each source of evidence, present characteristics for which data were charted and provide the citations. | 5-6 |
| Critical appraisal within sources of evidence | 16 | If done, present data on critical appraisal of included sources of evidence (see item 12). | N/A |
| Results of individual sources of evidence | 17 | For each included source of evidence, present the relevant data that were charted that relate to the review questions and objectives. | 6-9 |
| Synthesis of results | 18 | Summarize and/or present the charting results as they relate to the review questions and objectives. | 6-9 |
| **DISCUSSION** | | | |
| Summary of evidence | 19 | Summarize the main results (including an overview of concepts, themes, and types of evidence available), link to the review questions and objectives, and consider the relevance to key groups. | 9-13 |
| Limitations | 20 | Discuss the limitations of the scoping review process. | 11-12 |
| Conclusions | 21 | Provide a general interpretation of the results with respect to the review questions and objectives, as well as potential implications and/or next steps. | 13 |
| **FUNDING** | | | |
| Funding | 22 | Describe sources of funding for the included sources of evidence, as well as sources of funding for the scoping review. Describe the role of the funders of the scoping review. | 14 |
