# Supplementary material for: Use of Generative Artificial Intelligence in the Management of Low Back Pain: a Scoping Review
Source: J Med Syst. 2026 May 9;50(1):75. doi: 10.1007/s10916-026-02406-0 (PMC13157360; doi:10.1007/s10916-026-02406-0)
Supplement: Supplementary file 3 — Supplementary Material 3 [file 10916_2026_2406_MOESM3_ESM.docx]

# Appendix

### Detailed Search Strategy on Ovid-Medline

| 1 | exp Artificial Intelligence/  Artificial Intelligence.mp. |
| --- | --- |
| 2 | Deep Learning.mp. |
| 3 | Machine Learning.mp. |
| 4 | Supervised Machine Learning.mp. |
| 5 | Unsupervised Machine Learning.mp. |
| 6 | exp Telemedicine/  Telemedicine.mp. |
| 7 | exp Microcomputers/  Microcomputers.mp. |
| 8 | Smartphone.mp. |
| 9 | exp Mobile Applications/ Mobile Application*.mp. |
| 10 | Wearable Electronic Devices/ Wearable Electronic Device*.mp. |
| 11 | exp Digital Health/  Digital Health.mp. |
| 12 | exp Medical Informatics/  Medical Informatics.mp. |
| 13 | Natural Language Processing.mp. |
| 14 | exp neural networks, computer/  neural networks, computer.mp. |
| 15 | exp Image Processing, Computer-Assisted/  Image Processing, Computer-Assisted.mp. |
| 16 | Transformer*.mp. |
| 17 | Generative pretrained transformer.mp. |
| 18 | Fine-tuning.mp. |
| 19 | Pre-training.mp. |
| 20 | Embeddings.mp. |
| 21 | digital thera*.mp. |
| 22 | ChatGPT*.mp. |
| 23 | Large language model*.mp. |
| 24 | 1-23 (or) |
| 25 | exp Low Back Pain/  Low Back Pain.mp. |
| 26 | Sciatica.mp. |
| 27 | exp Sciatic Neuropathy/  Sciatic Neuropathy.mp. |
| 28 | lumbago.mp. |
| 29 | low* back pain.mp. |
| 30 | mechanical low* back pain.mp. |
| 31 | Back Pain/  Back Pain.mp. |
| 32 | backache.mp. |
| 33 | exp Back Injuries/  Back Injuries.mp. |
| 34 | (lumbar adj3 pain).mp. |
| 35 | exp Radiculopathy/  Radiculopathy.mp. |
| 36 | radicular syndrome.mp. |
| 37 | Nerve Compression Syndromes/  Nerve Compression Syndromes.mp. |
| 38 | piriformis muscle syndrome.mp. |
| 39 | exp Spondylosis/  Spondylosis.mp. |
| 40 | lumbar spondylosis.mp. |
| 41 | exp Spondylolisthesis/  Spondylolisthesis.mp. |
| 42 | 25-42 (or) |
| 43 | 24 AND 42 |
| 44 | limit 43 to yr="2012 -Current" |
